# Addressing Emotional Stress for Men Seeking Gynecological Care: A Call for Inclusivity and Sensitivity

**DOI:** 10.7759/cureus.64374

**Published:** 2024-07-11

**Authors:** Iason Psilopatis, Nikolaos Garmpis, Christos Damaskos

**Affiliations:** 1 Gynecology, University Hospital Erlangen, Erlangen, DEU; 2 Surgery, Sotiria Hospital, Athens, GRC; 3 Renal Transplantation Unit, Laiko General Hospital, Athens, GRC; 4 N.S. Christeas Laboratory of Experimental Surgery and Surgical Research, Medical School, National and Kapodistrian University of Athens, Athens, GRC

**Keywords:** medical racism, gynecologic malignancy, men’s health, primary breast malignancy, emotional problems

## Abstract

Men seeking gynecological care face unique emotional challenges due to societal norms and gender stereotypes. This work explores the emotional stress experienced by men in such situations, focusing on the discomfort stemming from traditional associations of gynecology with women's health. Despite these challenges, gynecological care is crucial for men, as it addresses conditions like prostate issues, pelvic pain, and sexually transmitted infections, which require specialized attention. The fear of judgment and societal expectations can deter men from seeking necessary medical attention, potentially leading to untreated health issues and poorer health outcomes. Proposed solutions to alleviate this stress include education campaigns, inclusive language, diverse healthcare providers, patient-centered communication, support groups, and ensuring privacy. By fostering inclusivity and awareness and prioritizing patient-centered care, healthcare systems can create a supportive environment where men feel comfortable seeking gynecological care, ultimately contributing to a more equitable healthcare system.

## Editorial

Introduction

In the realm of healthcare, the term "gynecologist" is traditionally associated with women's health, specifically focusing on the female reproductive system. However, men may find themselves in need of gynecological care, particularly when faced with conditions such as breast cancer. The societal stigma and discomfort surrounding men seeking gynecological attention can lead to significant emotional stress [1]. This article aims to explore the emotional challenges faced by men in such situations and propose concepts to foster a more inclusive and sensitive healthcare environment.

Understanding emotional stress

The emotional stress experienced by men seeking gynecological care stems from deeply ingrained societal norms and traditional gender roles. Men may feel a sense of vulnerability and discomfort due to the perceived incongruence between their gender identity and the traditionally female-associated nature of gynecology. The fear of judgment, embarrassment, and societal expectations can create barriers that hinder men from seeking timely and necessary medical attention [2].

Concepts to alleviate emotional stress

Education and Awareness Campaigns

Implementing educational initiatives and awareness campaigns can help debunk myths and misconceptions surrounding men's health, especially in the context of gynecological care. These campaigns can emphasize that men can develop conditions, such as breast cancer, that may require specialized medical attention [3].

Inclusive Language and Terminology

Healthcare providers and facilities should adopt more inclusive language to avoid reinforcing gender stereotypes. Using terms like "reproductive health specialist" or "breast health specialist" instead of exclusively relying on "gynecologist" can contribute to a more inclusive and welcoming environment [4].

Diverse Healthcare Providers

Encouraging diversity in the healthcare workforce can contribute to a more inclusive environment. Having male gynecologists or healthcare professionals specializing in reproductive health can help break down gender-related stereotypes and make men feel more comfortable seeking care.

Patient-Centered Communication

Healthcare professionals must prioritize patient-centered communication. This involves actively listening to patients, addressing concerns, and creating an open and non-judgmental space for dialogue. When discussing gynecological issues with men, providers should approach the conversation with sensitivity and empathy.

Support Groups and Counseling Services

Establishing support groups and counseling services specifically tailored for men facing gynecological concerns can provide a safe space for sharing experiences and addressing emotional stress. Connecting with others who have undergone similar experiences can reduce feelings of isolation and promote mental well-being [5].

Privacy and Confidentiality

Ensuring privacy and confidentiality in healthcare settings is crucial for building trust. Men seeking gynecological care should feel confident that their personal information and medical history are handled with the utmost discretion.

Conclusion

Addressing the emotional stress that men may experience when seeking gynecological care requires a multifaceted approach. By fostering inclusivity, promoting awareness, and prioritizing patient-centered care, healthcare providers can create an environment where men feel supported and comfortable seeking the medical attention they need. Breaking down gender-related stereotypes and challenging societal norms will contribute to a more equitable and empathetic healthcare system for everyone.
